# Factors influencing acceptability of voluntary HIV testing among pregnant women in Gamboma, Republic of Congo

**DOI:** 10.1186/s13104-015-1651-5

**Published:** 2015-11-06

**Authors:** Laure Stella Ghoma-Linguissi, Dagene Fruinovy Ebourombi, Anissa Sidibe, Thomas Serge Kivouele, Jeannhey Christevy Vouvoungui, Pierre Poulain, Francine Ntoumi

**Affiliations:** Fondation Congolaise pour la Recherche Médicale, Cité OMS, villa D6, Djoué, Brazzaville, Republic of Congo; Faculty of Health Sciences, Marien Ngouabi University, Brazzaville, Republic of Congo; Faculty of Sciences and Techniques, Marien Ngouabi University, Brazzaville, Republic of Congo; Institute for Tropical Medicine, University of Tübingen, Tübingen, Germany

**Keywords:** PMTCT, Women, HIV testing, Acceptability, Rural area, Republic of Congo

## Abstract

**Background:**

This study was carried out to identify factors affecting the acceptability of voluntary HIV testing among pregnant women in a semi-rural city, Gamboma, Republic of Congo.

**Methods:**

A cross-sectional study was conducted between January and September 2012. Pregnant women attending antenatal heath care at an integrated health center were enrolled after informed consent and followed through voluntary HIV testing.

**Results:**

Among 136 participants, 98 women (72 %) accepted voluntary HIV testing after pre-test counseling. Women with basic education, those who cited blood transfusion as a mode of transmission and prevention of mother-to-child transmission (MTCT) were more likely to accept testing as well those informed about free HIV testing. Interestingly, pregnant women who had heard about HIV/AIDS from hospital setting were less likely to accept testing.

**Conclusions:**

Our data indicate that increasing general education on HIV transmission/prevention modes is crucial for increasing acceptability of screening. Furthermore, HIV/AIDS knowledge disseminated to patients in hospital settings should be carefully monitored. Lastly, scaling-up MTCT services along with a better and larger community information, may address accessibility barriers observed in the present study.

## Background

Globally, 3.2 million children under 15 were living with HIV in 2013 [1] Over 700 children become newly infected with HIV each day [2]. The majority of children living with HIV were infected via mother-to-child transmission (MTCT), during pregnancy, labor, delivery or breastfeeding [3]. In comparison to other continents, Africa has the highest burden of the disease accounting for about 90 % of pediatric HIV infections [4].

Prevention of mother-to-child transmission (PMTCT) of HIV remains a top priority in HIV/AIDS disease control efforts. Mother-to-child transmission has been significantly reduced worldwide. In 2013, 16 % of children born to women living with HIV became infected, compared to 26 % in 2009 [1]. This decrease is particularly significant in high-income countries, where reduction of MTCT has been achieved through universal HIV screening of pregnant women and for those identified as HIV + through a three steps algorithm: (1) antiretroviral prophylaxis during pregnancy and labor, and antiretroviral ARV prophylaxis given to the infant in the first weeks of life; (2) obstetrical interventions including elective caesarean delivery (prior to onset of labor and membrane rupture); and (3) complete avoidance of breastfeeding [5–7]. Although complete avoidance of breastfeeding is an intervention of obvious utility, it may not be feasible in parts of the world where there is limited access to clean water and where there is no option than breastfeeding [8].

The World Health Organization (WHO) promotes a comprehensive approach to prevent MTCT based on four components [5, 9]. This approach includes (1) primary prevention of HIV among women at reproductive age; (2) preventing unintended pregnancies among women living with HIV; (3) preventing HIV transmission from a woman living with HIV to her infant through antiretroviral prophylaxis or treatment; and (4) providing appropriate treatment, care and support to mothers living with HIV and their children and families [10, 11].

The Republic of Congo (RoC) was one of the first countries in Africa to be affected by the HIV epidemic [12]. The HIV infection rate has declined among pregnant women in RoC from 3.4 % in 2009 to 2.8 % in 2012 as reported by the Congolese Ministry of Public Health. In comparison, HIV prevalence estimate was 3.2 % in the adult population in 2011 [13]. Regarding MTCT, limited data are available. In 1989, Lallemant et al. showed that, in Brazzaville, infant mortality in children born to women living with HIV exceeded 260 per 1000, while it was only 44 per 1000 in children born to HIV-negative mothers [14]. In 1994, the same team also demonstrated that the MTCT rate was of 40.4 % in Brazzaville [15]. These authors reported also an estimate MTCT rate of 25–40 % in Pointe–Noire [16]. The high transmission of HIV from mother to child in RoC, led to the implementation of a MTCT prevention policy in 2001.

A first strategic plan (2003–2007) focused on several objectives: reduction of 20 % of transmission rates; counseling and voluntary testing to all women during prenatal consultations; access to care and treatment, nutritional and psychological support for HIV positive mother and children [17]. The goal of the second strategic plan (2009–2013) in RoC aimed to improve the health of parents and their children by scaling up comprehensive PMTCT, paediatric HIV care and treatment, and support services. This resulted in major progresses with 18 % of newborns born to HIV positive women testing HIV positive, 22 comprehensive PMTCT trainings and the acceptability rate of voluntary HIV testing of 79.4 %.

As the national HIV/AIDS program of RoC aims to move from prevention to elimination of MTCT, one of the key indicators is the increase of acceptability of HIV testing in pregnant women. The present study conducted in the semi-rural city of Gamboma where health services have established PMTCT program, is the first to investigate factors that may influence the acceptance of HIV screening during pregnancy.

## Methods

### PMTCT program

The PMTCT program is based on the national guidelines of RoC. The entry point for pregnant women is integrated health centers (*Centre de Santé Intégré*, CSI) and the reference hospital.

PTMCT starts with health education session including introduction to the voluntary counseling and all participating pregnant women are proposed to be tested for HIV. After this group counseling, the counselors pursue with individual counseling for those who consented to be tested for HIV on the same day. Women who needed more time to think about the implication of this test could be screened later at their convenience.

Pre- and post-counseling were conducted by a trained health officer who administered the questionnaire. HIV screening was performed by an experienced technician and results were available within 30 min.

The investigator did not interfere with the normal procedure of the health facility, and was only present as an observer upon consent of study participant. The investigator was not present when the mid-wife provided the test result to the pregnant women. Pregnant women who were tested HIV positive, were referred to the local reference hospital where HIV health care including access to treatment and follow-up was provided.

### Study area and site

The study was conducted in Gamboma, a semi-rural city located in the center of RoC, in the department of Plateau, approximately 273 km from the capital city Brazzaville. It has an estimated population of forty three inhabitants with a reported HIV prevalence of 3.4 % in women and 2.9 % in men [18]. The study was carried out at the antenatal health care of the Gamboma І health facility. This center received the highest number of pregnant women for consultation and its medical staff had received formal training in PTMTC.

### Study design and population

A cross-sectional study was conducted. The recruitment was done between January and September 2012. A member of the medical staff presented the study to all pregnant women attending the health facility. The inclusion criteria for the study was all pregnant women who had not been tested for HIV during their current pregnancy and who consented to the study through signed informed consent.

### HIV testing

Following the national algorithm developed for the diagnosis of HIV in RoC, blood samples were tested using two rapid tests: Determine HIV 1/2 test (Alere GmbH, Koln, Germany) and ImmunoComb II HIV 1 & 2 BiSpot (Orgenics, Yavne, Israel). In case of discordance between these two tests, the enzyme-linked immunosorbent assay (ELISA, Vironostika^®^HIV-1 Plus O Microelisa System, UK) was performed by the reference laboratory in RoC which is the Laboratoire National de Santé Publique at Brazzaville as previously described by Linguissi et al. [19]. In this study, no serological discordant case was reported.

### Data collection and quality control

The following pieces of information were collected from the study participants using structured questionnaires: socio-demographic data (occupation, level of education, marital status), knowledge on HIV transmission, means of HIV prevention and acceptance of voluntary HIV screening. The study questionnaire was also translated and presented in two local languages (Lingala and Téké). Data coding and verification were performed by crosschecking the dataset at each step. The completeness and consistency of data was established by supervision of the principal investigator.

### Data processing

A total of 136 pregnant women were recruited in the study. Data were entered using software EpiInfo (version 3.5.3; Centers for Disease Control and Prevention, Atlanta, USA).

### Data analysis

Data were analyzed using SPSS software (version 16; IBM Corporation, New York, USA). Frequencies and proportions were used to describe the study population in relation to the relevant variables. Odds ratio, 95 % confidence interval and *p* value were computed to assess the presence and degree of association between acceptance of HIV testing and knowledge on HIV and MTCT. Logistic regression was the only statistic method used to compute that parameters. All statistical tests were significant when *p* value <0.05.

### Ethical statement

The study was approved by the Institutional Ethics Committee for Scientific Research and Health (CERSSA) (approval reference 00000067/DGRST/CERSSA) on the 30 March 2012. Permission to conduct the study was also obtained from the primary coordinator of the health worker in charge of the health facility. Women were informed about the nature of the study before enrolling in the study. If needed, discussions were translated in a local language (Lingala and Téké) by the investigators. The informed written consent of each woman was individually obtained and interviews were conducted in a place where the informant felt secure. The confidentiality of the discussion and of collected data was ensured. The interview was stopped at the convenience of the informant.

## Results

### Socio-demographic characteristics of the pregnant women

Table 1 shows socio-demographic characteristics of the 136 pregnant women enrolled in the study. The mean age was 25.5 ± 7.6 years within an interval ranging from 14 to 45 years. Approximately, 83 % of the women had a basic education level comprising of primary (34 %) and middle school (49 %). With regards to professional occupation, 39 % were farmer, 31 % housewives, 15 % saleswomen in markets and 13 % students. We also found that 63 % of the pregnant women were unmarried, 35 % were married or living as husband and wife, and 3 % were divorced or separated or widows.

**Table 1:** Influence of socio-demographic characteristics on acceptability of HIV testing in pregnant women, Gamboma, Republic of Congo

	Number of individual *N* (%)	Accept to do HIV test *N* (%)	Odds ratio (95 % CI)	*P* value
Age (years)				
14–24	67 (49)	47 (70)	Reference	
25–35	53 (39)	41 (77)	0.94 (0.45–1.94)	0.860
≥ 35	16 (12)	10 (63)	0.56 (0.18–1.69)	0.553
Education level				
No education	16 (12)	10 (63)	Reference	
Primary school	47 (34)	37 (79)	4.05 (1.13–14.43)	0.031*
Middle school	67 (49)	48 (72)	5.01 (1.46–17.33)	0.010*
High school	5 (4)	2 (40)	4.50 (0.54–37.38)	0.164
University	1 (1)	1 (100)	–	
Professional occupation				
Farmer	53 (39)	37 (70)	Reference	
Student	18 (13)	13 (72)	1.76 (0.59–5.24)	0.310
Housewife	42 (31)	33 (79)	2.24 (0.97–5.18)	0.059
Saleswoman	20 (15)	12 (60)	1.37 (0.48–5.17)	0.551
Others	3 (2)	3 (100)	2.24 (0.19–26.22)	0.521
Marital status				
Unmarried	85 (62)	64 (75)	Reference	
Married or cohabiting	47 (35)	31 (66)	1.30 (0.63–2.69)	0.551
Divorced or separated or widows	4 (3)	1 (75)	0.27 (0.03–2.70)	0.521

* statistically different

The influence of socio-demographic variables on the acceptance to voluntary HIV testing was assessed. No significant effect was observed between the age of pregnant women, professional occupation, marital situation and acceptance of HIV testing. However, pregnant women with primary and middle school education were four and five times more likely to accept HIV testing than women having no education.

### Knowledge of prevention and transmission modes of HIV/AIDS

From the 136 pregnant women recruited in this study, 98 (72 %) women accepted to be screened for HIV. Ninety-three percent of the women had heard about HIV/AIDS (Table 2). Pregnant women had heard of the disease mainly from parents/friends (73 %), within hospital settings or health facility (60 %) and in religious or school settings (32 %). Traditional media such as newspapers, books, radio and TV were less cited (26 % for radio and TV and 2 % for newspapers and books).

**Table 2:** Influence of knowledge of HIV/AIDS transmission/prevention modes on acceptability of HIV testing in pregnant women, Gamboma, Republic of Congo

	Number of individuals N (%)	Accept to do HIV test N (%)	Odds ratio (95 % CI)	*P* value
Heard of HIV/AIDS	127 (93)	91 (72)	0.63 (0.12–3.34)	0.590
Where did you hear about HIV/AIDS?				
Media (radio, TV)	35 (26)	28 (80)	1.77 (0.70–4.49)	0.228
News paper, books	3 (2)	2 (67)	0.77 (0.07–8.95)	0.847
Religion/school	43 (32)	34 (79)	1.71 (0.73–4.03)	0.218
Parents/friends	96 (71)	70 (73)	1.15 (0.51–2.60)	0.730
Hospital	68 (50)	41 (60)	0.29 (0.13–0.66)	0.003*
Mode of HIV/AIDS transmission				
Unprotected sexual intercourse	111 (82)	83 (75)	1.95 (0.77–4.91)	0.155
Contaminated objects	95 (70)	73 (75)	2.14 (0.97–3.41)	0.060
Blood transfusion	31 (23)	28 (90)	4.45 (1.26–15.73)	0.021*
MTCT	77 (57)	58 (75)	1.44 (0.67–3.07)	0.346
Prevention of HIV/AIDS				
PMTCT	58 (43)	47 (81)	2.37 (1.05–5.36)	0.038*
Condom	96 (71)	72 (75)	1.59 (0.70–3.59)	0.267
Fidelity	63 (46)	50 (79)	1.98 (0.90–4.33)	0.087
Abstinence	16 (12)	13 (81)	1.92 (0.51–7.27)	0.335
Avoid cutting objects	76 (56)	59 (78)	2.06 (0.94–4.51)	0.070
Knowledge of free HIV testing	90 (66)	77 (86)	6.99 (3.02–16.19)	P<0.001*
Knowledge of the existence of a HIV testing center	70 (51)	58 (83)	3.06 (1.37–6.79)	0.006*

* statistically different

With regards to the modes of HIV transmission, unprotected sexual intercourse was cited among 82 % of the women, followed by contaminated objects (70 %) and MTCT (57 %). Blood transfusion was cited last with 23 %.

The most cited prevention modes of contamination were condoms use (71 %), avoid cutting objects (56 %), fidelity between partners (46 %) and PMTCT (43 %). Abstinence was at the bottom of the list (12 %).

Women who have been informed of HIV at the hospital or health facility were three times less likely to accept HIV testing. Among the modes of HIV transmission, pregnant women informed about the risk to get HIV through blood transfusion were four times more likely to accept HIV testing. Pregnant women who cited the PMTCT program among one of prevention modes of HIV transmission were two times more likely to accept HIV testing.

As shown in Table 2, pregnant women who knew HIV testing was free of charge represented 66 % of all participants and were seven times more likely to accept HIV testing. With regards to the existence of a health care center, 51 % knew its existence. These women were three times more likely to accept HIV testing.

## Discussion

The present study is the first to report the acceptability of voluntary HIV testing among pregnant women in a rural city, Gamboma, in RoC. We found that 72 % of pregnant women accepted HIV testing. This observation is comparable to what is observed in other sub-Saharan African countries [20]. Albeit encouraging, a higher acceptance of HIV testing among pregnant women is crucial if one wants to eliminate new HIV infections among infants.

Given that we observed an HIV prevalence of 11 % among pregnant women in Gamboma, this means that among the 38 women who refused HIV screening, four women might have been HIV infected and missed for PMTCT interventions. It is plausible that women who refused HIV testing refused it because they knew or believed they had behavioral risks and therefore might be infected. Hence the proportion of missed opportunities for PMTCT could be higher than what has been calculated on the basis of 11 % HIV prevalence in Gamboma. To further increase the coverage of voluntary HIV testing, the opt-out approach for HIV-testing at antenatal care services may be considered as several African countries already adopted it [20].

The socio-demographic characteristic of the pregnant women recruited in the study is reminiscent to that observed in similar studies conducted in rural African settings [21–23], characterized by a low level of education and farming as the primary occupation. Approximately 2/3 of the pregnant women in our cohort were unmarried but no association was found between marital status and the acceptability of HIV testing, even though previous studies including one conducted in RoC [24] and in South Africa [25] reported an association between HIV incidence and marital status.

Pregnant women who attended primary or middle school accepted HIV test up to five times more than women with no education. The group of “high school or university” was too small to allow any interpretation regarding the influence of education level on the acceptance of HIV test. However, education plays a key role in the understanding of HIV counseling and has been found to be associated with Voluntary Counseling and Test (VCT) acceptance [26–29].

In the present work, our findings showed that 93 % of pregnant women knew that HIV/AIDS was a transmissible disease. We noticed that women were less informed about PMTCT then unprotected sexual transmission suggesting that specific effort should be done by the national HIV control program towards women, for example through community based approaches [30, 31].

The most interesting finding was the association observed between the source of HIV/AIDS information and the acceptability to HIV testing. Pregnant women who had heard of the disease in hospital and health facilities were three times less likely to accept HIV testing. One explanation could be a relative fear regarding hospitalization or a psychological disturbance related to admission in a health facility. Also the way counseling is performed could be another explanation. Issues surrounding counseling are known to represent a major challenge in HIV test acceptance [32, 33]. In the case of PMTCT, the quality of pre-counselling is a key factor influencing the acceptance of HIV testing among pregnant women [34, 35].

It is important to highlight the fact that this study has been conducted in rural health facilities, therefore the findings may not be generalized to urban facilities. There are significant differences in the socio-demographic structure of populations that live in urban versus rural areas, with urban population being more educated and economically advantaged compared to rural population [36, 37].

To reduce MTCT, mechanisms to increase participation in HIV testing among pregnant women are essential. Firstly, it is important to raise awareness about the good efficiency of PMTCT. Secondly, it would be desirable to involve men or partners to participate in prenatal visits in order to limit the fear of rejection or discrimination.

One limitation of this study was that interviews were conducted by the same midwives who performed voluntary counseling and test (VCT). This may have influenced results, and precluded analysis of whether characteristics of the pre-test counselling received affected acceptance rates. Another limitation is the small sample size of the cohort albeit the enrollment period ran nine months. Notwithstanding these limitations, we believe that our study has very important findings for strengthening PMTCT implementation in Gamboma and in the country.

The current HIV/AIDS campaign momentum in the RoC should be strengthened. More media or radio talks should be aired to educate families and community members on HIV/AIDS issues. The community action to reduce stigma around HIV/AIDS should be encouraged. The pregnant women’s partner involvement in the process of HIV counseling and testing is essential if pregnant women are expected to fully participate and benefit from PMTCT programs.

## Conclusion

This first study conducted among pregnant women in a semi-rural area of the Republic of Congo showed that level of education and general knowledge of HIV transmission/prevention are barriers to the acceptability of voluntary HIV testing. There is a need to scaling up mass information targeting this specific population for ensuring that newborns will be protected from the infection.
